# Comment to: Face mask use and viral load in patients with mild symptoms of COVID-19

**DOI:** 10.31744/einstein_journal/2025CE1711

**Published:** 2025-07-23

**Authors:** Salvatore Chirumbolo

**Affiliations:** 1 University of Verona Department of Engineering for Innovation Medicine Verona Italy Department of Engineering for Innovation Medicine, University of Verona, Verona, Italy.

Dear Editor,

A recent study by Costa et al.^(1)^ investigated the association between facemask usage and SARS-CoV-2 viral load in individuals with mild COVID-19 symptoms. This cross-sectional study, conducted at a public emergency care facility in Belo Horizonte, Brazil, aimed to explore whether the regular use of facemasks is correlated with lower viral loads in infected individuals. The principal findings revealed a significantly lower probability of high SARS-CoV-2 viral load in individuals who regularly wore masks than those who did not, thereby prompting further discussion within the scientific community.^(2)^

A significant strength of this study is its timely investigation of a pressing public health concern, providing empirical evidence supporting the use of facemasks beyond their conventional role in preventing infection. While several studies have confirmed that masks lower the risk of SARS-CoV-2 transmission, there is limited research examining their effect on the viral load in individuals who contract the virus.^(3)^ The investigation builds upon existing literature and employs an observational methodology that adheres to ethical constraints, as conducting randomized controlled trials on mask usage and infection severity remains impractical.^(1)^

The study employed RT-PCR to quantify viral loads and categorized the participants into regular and non-regular mask users based on their self-reported mask-wearing frequency. Those who reported wearing masks "every time" or "most of the time" were designated as regular users, while those who indicated wearing masks "sometimes," "rarely," or "never" were considered as non-regular users. The principal findings revealed that within the first five days post-symptom onset, 64.9 % of non-regular mask users had a high viral load, in contrast to 45.5 % of regular mask users (p=0.0073). This pattern persisted in subsequent periods, with non-regular mask users consistently presenting with higher viral loads. The authors identified a statistically significant difference in viral loads for up to eight days post-symptom onset, beyond which the differences between the groups became less pronounced.

Despite these strengths, this study had several limitations that should be considered when interpreting the results.

The reliance on self-reported data concerning mask usage introduces the potential for recall flawsand social desirability biases. Participants may have exaggerated their compliance with mask-wearing protocols, particularly in light of public health campaigns advocating mask use during the pandemic. Additionally, this study did not account for variations among different mask types, such as cloth masks, surgical masks, and N95 respirators, which may have differing levels of effectiveness in minimizing viral load exposure.

A primary limitation of this study was its cross-sectional design, which restricts the ability to establish causal links . Although the study found a correlation between mask usage and decreased viral loads, it remains uncertain whether mask-wearing directly influences viral load or if other confounding factors are involved. For instance, individuals who regularly wore masks might also have adopted additional preventive measures, such as maintaining social distancing, adhering to enhanced hygiene protocols, or steering clear of high-risk settings, which could have contributed to the observed variations in viral load.

This study encountered methodological challenges when defining high and low viral loads. The authors used a cycle threshold (Ct) value of 20 as an arbitrary cut-off for viral load classification. Although the authors acknowledge that Ct values are subject to fluctuations, and that there is no universally accepted threshold for distinguishing between high and low viral loads, the decision to use 20 may have influenced the results. Conducting sensitivity analyses with alternative Ct cutoffs could enhance the conclusions of the study by demonstrating the robustness of the findings.

The statistical approach adopted by Costa et al. raised significant concerns related to the sample size and generalizability of the results. The study comprised 441 individuals who tested positive by RT-PCR, of whom only 54 were identified as non-regular mask users, leading to wider confidence intervals and reduced statistical power. Additionally, the study cohort was exclusively sourced from a single emergency care facility in Belo Horizonte, Brazil, which may not adequately represent the characteristics of broader populations with diverse demographic, geographic, and socioeconomic backgrounds.

These findings corroborate the hypothesis that mask usage mitigates viral exposure, potentially resulting in lower viral loads during infection. This observation aligns with the "inoculum effect" theory, which suggests that lower initial viral exposure could lead to less severe disease manifestations. Nonetheless, it is crucial to acknowledge that the study was confined to participants with mild symptoms, and thus did not evaluate whether lower viral loads were associated with less severe disease or improved clinical outcomes. Future investigations should aim to determine whether reduced viral loads attributable to mask usage correlate with decreased hospitalization rates, a lower risk of prolonged COVID-19, and expedited recovery periods.

The findings of this study support public health directives advocating mask-wearing, particularly in high-transmission settings. Should mask usage correlate with reduced viral loads, it could have implications that extend beyond infection prevention and potentially mitigate disease severity and subsequent transmission. This is particularly significant, given the emergence of SARS-CoV-2 variants with diverse transmissibility and immune evasion.

Future research should aim to address the limitations of this study. Longitudinal studies tracking viral load progression among infected individuals with differing mask-wearing practices would provide more compelling evidence of a causal relationship.^(4,5)^ Additionally, studies accounting for confounding variables such as social distancing, ventilation, and vaccination status would help isolate the specific effect of mask usage on viral load. Comparative research on various mask types and their fit quality could further refine the public health recommendations regarding optimal mask usage.

In conclusion, this study provides significant insights into the correlation between mask usage and the SARS-CoV-2 viral load. These findings support the hypothesis that regular mask-wearing is associated with lower viral load. However, the methodological limitations highlight the need for further research. These results emphasize the importance of facemasks as a preventive measure, not only in reducing infection risk but also in potentially mitigating viral exposure and disease severity. Nonetheless, it is imperative for policymakers and public health officials to interpret these findings with caution, acknowledging the observational nature of the study and the potential biases. Addressing these limitations through a more robust study design is crucial for advancing our understanding of the protective benefits of facemasks in controlling infectious diseases.
